# Timed sulfonylurea modulation improves locomotor and sensory dysfunction following spinal cord injury

**DOI:** 10.3389/fphar.2024.1440198

**Published:** 2024-08-01

**Authors:** Guo-Ying Xu, Manjit Maskey, Zizhen Wu, Qing Yang

**Affiliations:** Department of Neurobiology, University of Texas Medical Branch, Galveston, TX, United States

**Keywords:** spinal cord injury, ATP-gated potassium channels, diazoxide, glibenclamide, chronic pain

## Abstract

Traumatic spinal cord injury (SCI) results in immediate tissue necrosis and delayed secondary expansion of neurological damage, often resulting in lifelong paralysis, neurosensory dysfunction, and chronic pain. Progressive hemorrhagic necrosis (PHN) and excessive excitation are the main sources of secondary neural injury. Recent approaches to attenuate PHN by glibenclamide can improve locomotor function after SCI. However, use of glibenclamide can exacerbate development of SCI-induced chronic pain by inhibiting K_ATP_ channels to increase neuronal excitation and glial activation. In this study, we explored a treatment strategy involving administration of glibenclamide, which suppresses PHN, and diazoxide, which protects against neuronal excitation and inflammation, at different time intervals following spinal cord contusion. Our goal was to determine whether this combined approach enhances both sensory and motor function. Contusive SCI was induced at spinal segment T10 in adult rats. We found that K_ATP_ channels opener, diazoxide, decreased the hyperexcitability of primary sensory neurons after SCI by electrophysiology. Timed application of glibenclamide and diazoxide following contusion significantly improved locomotor function and mitigated development of SCI-induced chronic pain, as shown by behavioral evidence. Finally, we found that timed application of glibenclamide and diazoxide attenuates the inflammatory activity in the spinal cord and increases the survival of spinal matters following SCI. These preclinical studies introduce a promising potential treatment strategy to address SCI-induced dysfunction.

## 1 Introduction

Spinal cord injury (SCI) is a devastating condition often resulting from sudden traumatic impacts such as accidents or acts of violence. These injuries can lead to a cascade of complex pathophysiological events, significantly affecting the quality of life. The initial mechanical insult, termed the primary injury, causes immediate damage to blood vessels and neuronal tissues. However, many neuronal axons and interneurons survive this initial trauma but remain vulnerable to further damage from secondary degeneration processes (Hu et al., 2023). This secondary phase is characterized by a series of biochemical and cellular events that exacerbate the initial injury, leading to progressive neuronal necrosis and expansion of the lesion. Understanding and intervening in these secondary cascades are crucial for improving outcomes post-SCI.

Among the many consequences of secondary injury, progressive hemorrhagic necrosis (PHN) is particularly noteworthy. PHN contributes significantly to the worsening of spinal cord damage after the primary injury. The molecular mechanisms driving PHN involve the upregulation and interaction of transient receptor potential melastatin 4 (TRPM4) channels with sulfonylurea receptor 1 (SUR1) in the endothelial cells of capillaries (Simard et al., 2007; Gerzanich et al., 2009; Woo et al., 2013). This pathological interaction disrupts cellular homeostasis and leads to extensive hemorrhagic damage.

In recent years, therapeutic strategies targeting these molecular pathways have shown promise. Notably, the SUR1 subunit regulator glibenclamide has been found to reduce hemorrhagic necrosis and improve locomotor outcomes when administered immediately after SCI. However, its use is not without drawbacks. While glibenclamide’s ability to decrease hemorrhagic necrosis and enhance motor function is well-documented (Simard et al., 2007; Popovich et al., 2012; Simard et al., 2012), it paradoxically worsens sensory functions post-SCI, likely due to its effects on glial cell activation (Redondo-Castro et al., 2013).

Conversely, diazoxide, a selective opener of SUR-K_ATP_ channels, has shown potential in protecting against neurodegeneration and reducing glial activation following SCI (Roseborough et al., 2006; Virgili et al., 2013; Martinez-Moreno et al., 2016). These findings suggest that targeting SUR subunits with precise timing and specific agents could be a strategic approach to mitigate the deleterious effects of SCI. By carefully modulating these pathways, it might be possible to enhance motor recovery while preventing the onset of chronic pain and other sensory deficits.

Therefore, here we investigated the therapeutic potential of manipulating SUR subunit activity using agonists and antagonists at distinct time points after SCI. By evaluating the effects of timed administration of glibenclamide and diazoxide, we seek to determine whether this approach can optimize outcomes for both motor and sensory functions. Our findings suggest that a tailored regimen of these treatments can significantly improve locomotor function and alleviate the development of chronic pain induced by SCI, providing a promising therapeutic strategy for managing SCI-related dysfunctions.

## 2 Methods

This study adhered to guidelines set forth by the National Institutes of Health and received approval from the Institutional Animal Care and Use Committee at the University of Texas Medical Branch in Galveston. We acquired adult Sprague-Dawley rats weighing 200–250 g from Charles River Laboratories (Wilmington, MA). Both male and female rats were included in the study, and no noticeable differences related to sex were observed. Animals were housed two per cage in a temperature-controlled room with a 12-h light/dark cycle, maintaining a temperature of 21°C ± 1°C. Animals had unrestricted access to food and water throughout the study. Prior to commencement of the experiments, rats were allowed to acclimate to their surroundings for 1 week.

### 2.1 Spinal cord injury (SCI)

SCI in humans typically arises from spinal cord contusion, so we used a spinal cord contusion model. Recognizing variability in the extent of spinal cord damage resulting from different contusion models (Young, 2002), we used an Infinite Horizon impactor (Precision Systems and Instrumentation, Lexington, KY) because this device reliably simulates contusive injury by rapidly applying a defined force (Scheff et al., 2003).

Experimental animals were anesthetized using a combination of acepromazine (0.75 mg/kg), xylazine (20 mg/kg), and ketamine (80 mg/kg). A laminectomy was performed, followed by induction of a moderate spinal contusion at T10 using a force of 150 kdyne and a 1-s dwell time. In contrast, the sham group only had backbone removal without spinal cord contusion. Following the procedure, the muscles were stitched over the backbone, and the skin was closed with clips. Animals were then placed in cages equipped with heating pads set at ∼37°C. Animals received analgesic injections (buprenorphine; 0.02 mg/kg intraperitoneally) twice daily for 5 days, along with prophylactic antibiotics (Baytril, 2.5 mg/kg intraperitoneally) for 10 days. Manual bladder evacuation was performed twice daily until signs of bladder function recovery were observed. Regular monitoring of the rats was carried out to detect any signs of severe pain, such as excessive grooming, pronounced inactivity, or self-mutilation. Any animals that showed severe signs of pain were euthanized immediately.

### 2.2 Dorsal root ganglion (DRG) neuron dissociation and culturing

Rats were deeply anesthetized with Beuthanasia (75 mg/kg, Merck Animal Health, Kenilworth, NJ), followed by intracardial perfusion of cold Dulbecco’s phosphate-buffered saline (DPBS, without calcium and magnesium). Ganglia were dissected from lumbar vertebrae 4 and 5 (L4/L5). L4/L5 DRGs were minced with fine scissors and placed in Dulbecco’s Modified Eagle Medium (DMEM; Invitrogen, Waltham, MA) containing 0.35 mg/mL trypsin (Worthington, Lakewood, NJ) and 0.8 mg/mL collagenase (Roche, Mannheim, Germany) for 40 min at 34°C. The cell-containing solution was centrifuged (300 rpm for 5 min) and resuspended. Isolated cells were plated onto 8-mm coverslips (Warner Instruments, Hamden, CT) precoated with 50 mg/mL poly-L-lysine and incubated in DMEM without serum overnight (37°C, 5% CO_2_).

### 2.3 Electrophysiological recordings

Electrodes were fashioned from borosilicate glass capillaries (BF150-110-10, Sutter Instrument Co., Novato, CA) using a P-1000 micropipette puller (Sutter Instrument Co., Novato, CA), ensuring a resistance of ∼2 MΩ for subsequent experiments. Neurons were observed with an Eclipse Ti inverted microscope (Nikon, Tokyo, Japan). The junction potential was around 5.0 mV and was electronically compensated. Whole-cell configuration was established by patching cells at -60 mV using a MultiClamp 700B amplifier with an Axon Digidata 1550B data acquisition system (Molecular Devices, San Jose, CA). The cell membrane capacitance (20.5±1.6 pF) and series resistance (4.9±0.4 MΩ) were compensated before switching to whole-cell current clamp. Resting membrane potential and any spontaneous DRG firing were recorded by patching cells at 0 pA for 15 s in a normal external solution, followed by local application of 100 μM diazoxide for 1 min. Signals were sampled at a rate of ≥10 kHz. The pipette solution contained: 134 mM KCl, 1.8 mM CaCl_2_, 13.2 mM NaCl, 1.6 mM MgCl_2_, 9 mM HEPES, 3 mM EGTA, 1 mM Mg-ATP, and 0.3 mM Na-GTP (pH 7.2, 300 mOsM). The bath solution contained: 140 mM NaCl, 1.8 mM CaCl_2_, 3 mM KCl, 2 mM MgCl_2_, 10 mM glucose, and 10 mM HEPES (pH 7.4, 320 mOsM). The osmolality of both external (bath solution) and internal (pipette solution) solutions were measure three times by Wescor Vapro Pressure Osmometer (Model 5600, ELITechGroup Inc, Logan, UT). The pipette solution was aliquoted and stored at -80°C for up to 6 months. The bath solution was kept at 4°C for no more than 2 weeks.

The exchange of solutions was achieved by rapidly shifting the array of fused silica columns (inner diameter, 200 µm) horizontally using a micromanipulator. One end of each silica column was connected to a series of independent syringes containing extracellular solution, either with or without 100 µM diazoxide. The open ends of the silica columns were positioned approximately 100 µm from the cells under observation. The solutions were delivered to the cells by gravity.

### 2.4 Delivery of glibenclamide and/or diazoxide

It has been reported that blocking SUR1 with glibenclamide immediately after spinal contusion for 24 h reduces hemorrhagic necrosis and leads to long-term enhancements in locomotion and coordination (Simard et al., 2007; Popovich et al., 2012; Simard et al., 2012). Therefore, glibenclamide was administered acutely for 24 h using an intradermally implanted mini-osmotic pump (200 ng/0.5 μL/h; Alzet Corp., Cupertino, CA), beginning 30 min after impact. This timing aligns with the most pronounced hemorrhage occurring at 3–24 h after acute SCI (Noble and Wrathall, 1989). Hemorrhage usually persists in the lesion site for up to 6 days (Noble and Wrathall, 1989), Thus, we also applied glibenclamide for 6 days, starting 30 min after the contusion. Subsequently, animals received systemic diazoxide treatment (5 mg/kg, twice/day) as indicated in each experiment.

### 2.5 Behavioral tests

*Pain-like reflex sensitivity tests:* Behavioral tests were assessed during the light phase of the day before and after SCI/sham. Personnel conducting behavioral tests were unaware of the drug treatments administered. To acclimatize to the testing chamber, animals spent 20 min in the chamber before experiments commenced. We used a series of calibrated von Frey filaments (Stoelting, Wood Dale, IL), which were applied to the hairless surface of the hindpaws. Mechanical sensitivity thresholds were determined using the “up-down” method (Chaplan et al., 1994).

*Conditioned place preference (CPP) test:* To evaluate spontaneous pain, we used a CPP device (Med Associates Inc., Georgia, VT) (Yang et al., 2014; Wu et al., 2020) consisting of one white chamber and one black chamber. During the initial day, each animal underwent a 30-min habituation period in the CPP chambers. Over the subsequent 3 days, animals were conditioned with twice-daily injections of analgesic and vehicle, each associated with a specific chamber. In the analgesic conditioning session, the rat received an intraperitoneal injection of retigabine (10 mg/kg) and was placed in the white chamber for 1 hour, beginning 5 min after injection. During a distinct conditioning session, the identical rat was isolated within the black chamber for 1 h after receiving an intraperitoneal saline injection. On the fifth day, the test animal, without receiving any injections, was positioned inside the black chamber and allowed unrestricted access to both chambers. The system monitored duration of time the animal spent in black and white chambers over 30 min.

*Basso, Beattie, and Bresnahan (BBB) locomotor scoring:* To assess locomotor function, we used the 21-point BBB locomotor test (Basso et al., 1996). Animals were positioned within an open field enclosure (1.1 m diameter), and their locomotor behavior was observed and evaluated under white light conditions. Joint and limb movements, paw placement and stepping, coordination, as well as trunk stability and tail positioning, were scored on the 21-point (0–20) rating scale (Basso et al., 1996). The testing procedure was carried out daily for the initial 5 days and subsequently on a weekly basis for 5–6 weeks post-contusion. Rats with a BBB score of ≥1 on the first day following SCI/sham were excluded for analysis.

*Horizontal ladder*: BBB scores typically peak 3 weeks post-SCI. In contrast, the horizontal ladder test reveals that locomotor function continues to improve until week 6 post-SCI (James et al., 2011). We thus also employed horizontal ladder test in this study. One week prior to SCI/sham, a training regimen was implemented. During this training, rats were daily coached to traverse a 1-m-long horizontal ladder with a testing section spanning 0.8 m. Within the testing area, there were 10 rungs randomly spaced 3–8 cm apart. Each training session encompassed three ladder crossings. On the conclusive day of the training period, tests were recorded on video. Videos were reviewed in slow motion to quantify hindlimb misses and footslips throughout the crossings. This evaluation allowed assignment of a score to each tested animal, with a score of 1 allocated when an entire paw descended below a rung during testing. This assessment was repeated once weekly for 3 weeks, starting on day 21 following SCI/sham.

### 2.6 Spectrophotometric assay for spinal cord hemorrhage

Hemoglobin content in the spinal cord following SCI was quantified using a spectrophotometric assay, following a previously described method (Choudhri et al., 1997). Rats were euthanized using Beuthanasia (Merck, Kenilworth, NJ) and perfused with cold heparinized PBS to remove intravascular blood. Spinal cord segments spanning the lesion site (5 mm) were dissected. Each sample was mixed with 250 μL of distilled water, homogenized for 30 s, and sonicated for 1 min (Qsonica, Newtown, CT) on ice. Homogenized samples were centrifuged at 13,000 rpm for 30 min, and the supernatant containing hemoglobin was collected.

For quantification, 80 µL of Drabkin’s reagent (RICCA Chemical, 266,016) was added to 20 µL of sample and incubated for 15 min to fully convert hemoglobin to cyanomethemoglobin. This product was then assessed using an iMark Microplate Reader (Bio-Rad, Hercules, CA) at ∼540 nm wavelength (OD1). As an additional control measure, blood from a naïve rat was collected via cardiac puncture after anesthesia, and 1 µL of blood was added to 20 µL of spinal cord specimen collected from the naïve rat for measurement (OD2). Optical density of all experimental samples was normalized to that of the naïve specimen. Blood volume (µL) from each experimental spinal cord following SCI was calculated as: (OD1/OD2) x (250/20).

### 2.7 Western blotting

After completing behavioral tests, animals underwent deep anesthesia with Beuthanasia followed by perfusion with ice-cold PBS. Subsequently, L4/L5 segments of the spinal cord from each rat were extracted and placed into 1.5-mL Eppendorf tubes on dry ice. Tissues were homogenized in 500 μL of RIPA lysis buffer (#R0278, Sigma-Aldrich, St. Louis, MO), and protease inhibitor cocktail (#PI78430, Thermo Fisher Scientific, Waltham, MA) was added. Following homogenization, samples underwent three rounds of sonication (10-s pulses) and centrifugation (14,000 rpm for 10 min) at 4°C. Total protein concentration of the lysates was measured by Nanodrop One (Thermo Fisher Scientific). Samples were prepared for SDS-PAGE (Bio-Rad, 4%–20% Tris-HCl) by diluting in 1:1 with Laemmli sample buffer, and 30 μg of proteins were loaded into each well. Following electrophoresis, gel contents were transferred onto PVDF membranes using a Bio-Rad Turbo Transfer system. Membranes were blocked with 5% nonfat dry milk in 0.1% Tween-20/TBS and incubated overnight at 4°C with antibodies against GFAP (AB5541, Millipore, Burlington, MA), Iba-1 (019-19741, Wako, Richmond, VA), and β-actin (ab8226, Abcam). Membranes were then incubated with horseradish peroxidase-conjugated anti-rabbit or anti-mouse IgG (Jackson ImmnuoResearch, West Grove, PA) for 1 h at room temperature and developed using an enhanced chemiluminescence kit (Pierce, Waltham, MA). Protein expression levels were quantified by measuring optical density using NIH ImageJ software. Molecular weight standards were run on each gel, and β-actin served as the loading control.

### 2.8 Eriochrome cyanine (EC) histochemistry

To distinguish between gray and white matter in spinal cord sections, we used iron EC staining (Rabchevsky et al., 2001; James et al., 2011). After completion of all behavioral tests (42 days post-SCI), animals were humanely euthanized using Beuthanasia (Merck, Kenilworth, NJ) and perfused with cold PBS, followed by a 4% PFA. Spinal cords, with the lesion site positioned centrally (10 mm in length), were extracted and post-fixed in 4% PFA overnight. Tissues were then immersed in 30% sucrose at 4°C. Spinal cords at the injury site were encased in cryo-embedding medium (OCT, Sakura Finetek, Torrance, CA) and preserved at −80°C. Tissues were sliced into 20-μm-thick transverse sections on an HM525 NX Cryostat (Epredia, Kalamazoo, MI). Separation of 200 μm was maintained between sections. The particular section for each animal that exhibited the most substantial cavity was designated as the lesion epicenter. Assessment of the lesion volume spanned 5 mm caudally to rostrally starting from the epicenter. Sections were subjected to a sequence of procedures (Wu et al., 2020), including dehydration, clearing, rehydration, and staining. Stained sections were visualized on a Nikon microscope and analyzed with Nikon NIS-Elements software.

### 2.9 Statistics

Sigmaplot (version 14, Systat software, San Jose, CA) and Prism (version 9.0, GraphPad, La Jolla, CA) were used for data analysis. Data are presented either as mean ± standard error of the mean or as percentages. A significance level (α) of 0.05 was established for all statistical tests. When comparing more than two groups, we applied either one-way or two-way ANOVA, followed by Tukey’s multiple comparison tests. For comparisons within the same cohort before and after treatment, two-tailed paired t-tests were conducted. *p* < 0.05 was considered statistically significant.

## 3 Results

### 3.1 Diazoxide decreases hyperexcitability of primary sensory neurons induced by SCI

**FIGURE 1:**
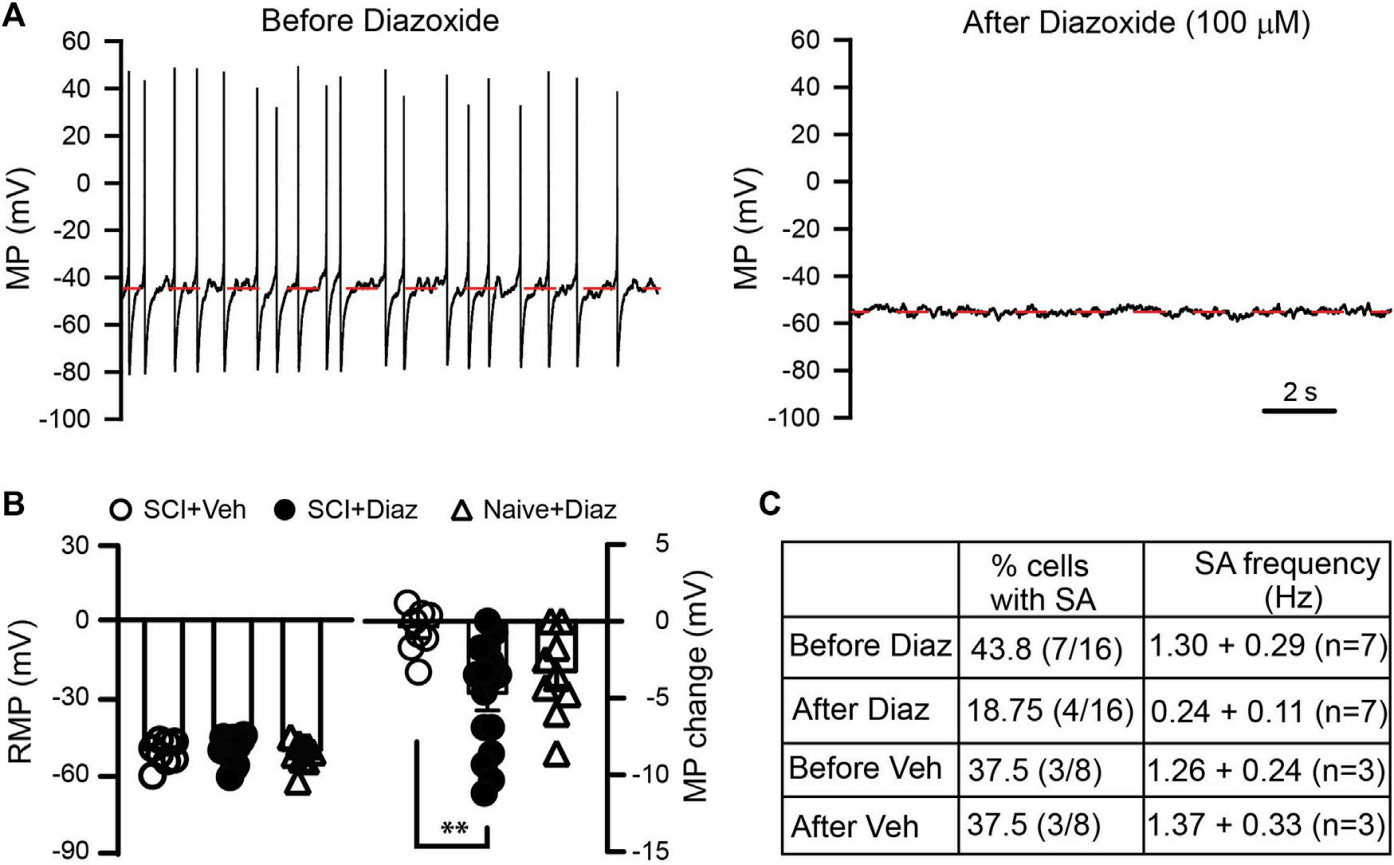
*Effect of diazoxide on the hyperexcitability of dorsal root ganglion (DRG* *) neurons induced by SCI.* **(A)** Representative recordings showing that spontaneous activity of DRG neurons from a contusive animal were largely blocked after cells were exposed to diazoxide for 1 min. Note that cell membrane potential is hyperpolarized from about −46 mV to −53 mV. **(B)** Summary data indicate the baseline membrane potential before vehicle (Veh) or diazoxide (Diaz) application (left panel), as well as changes in membrane potential upon local application of vehicle or diazoxide (100 μM). Each circle represents one DRG neuron. **p < 0.05, One way ANOVA. **(C)** Table indicates the effects of diazoxide on firing of DRG neurons from SCI rats.

After SCI, heightened activity of primary sensory neurons plays a crucial role in chronic pain development and maintenance (Bedi et al., 2010; Yang et al., 2014; Wu et al., 2020). Since potassium (K^+^) channels are pivotal in regulating neuronal excitability, we initially investigated whether diazoxide could mitigate the excitability of L4/L5 DRG neurons from SCI rats. Most cells exhibited a more negative membrane potential after diazoxide treatment compared to vehicle controls, and diazoxide greatly decreased the firing rate of neurons that initially firing spontaneously (Figures 1A–C). These results indicate that diazoxide effectively decreased the excitability of DRG neurons induced by SCI. In addition, we also detected effect of diazoxide on membrane potential of naïve DRG neurons. There is no significant difference in diazoxide-induced membrane shift between SCI and naïve groups (Figure 1B).

### 3.2 Diazoxide reduces chronic hypersensitivity of hindpaws following SCI

**FIGURE 2:**
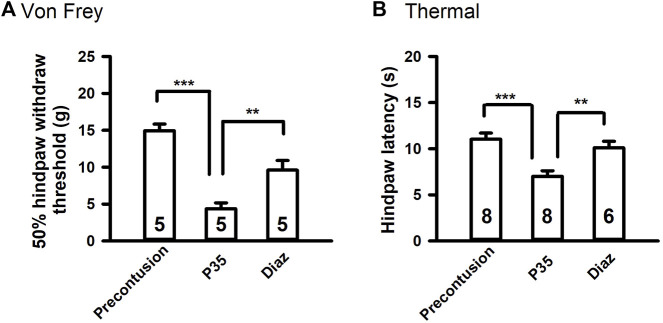
Diazoxide attenuates chronic pain-like behaviors in SCI rats. **(A)** Effects of diazoxide (Diaz, 5 mg/kg, i. p.) on chronic SCI-induced mechanical hypersensitivity tested 120 min after injections. **(B)** Effects of diazoxide on chronic thermal hypersensitivity following SCI. Animal numbers are indicated on columns. **p < 0.01, ***p < 0.001, one-way ANOVA followed by Tukey’s *post hoc* test.

Since diazoxide decreases the excitability of primary sensory neurons, we investigated whether diazoxide reduces SCI-induced chronic pain. Before contusion, each animal underwent behavioral pretests involving mechanical and heat stimuli applied to the plantar surface of each hind paw. Five weeks after contusion, the SCI (spinal cord injury) animals received same mechanical and thermal tests before and after the injection of diazoxide (5 mg/kg, i. p.). It is consistent with our previous report that spinal cord contusion significantly increased reflex sensitivity to both mechanical and heat test stimuli. Diazoxide injection significantly reversed both mechanical and thermal hypersentivity (Figure 2). Diazoxide-induced effect in SCI animals was observed 1–2 h after i. p. injection, and disappeared after 18 h (overnight, data not show).

### 3.3 Diazoxide attenuates development of chronic pain following SCI

Acute application of glibemclamide exacerbates sensory dysfunction by affecting glial cell activation (Redondo-Castro et al., 2013). Both neuronal hyperexcitation and glial activation contribute to secondary degeneration and development of neuropathic pain after SCI (Hains and Waxman, 2006; Tan et al., 2009; Yang et al., 2014; Wu et al., 2020). Therefore, we investigated whether diazoxide improved sensory dysfunction after SCI with or without glibenclamide treatment.

**FIGURE 3:**
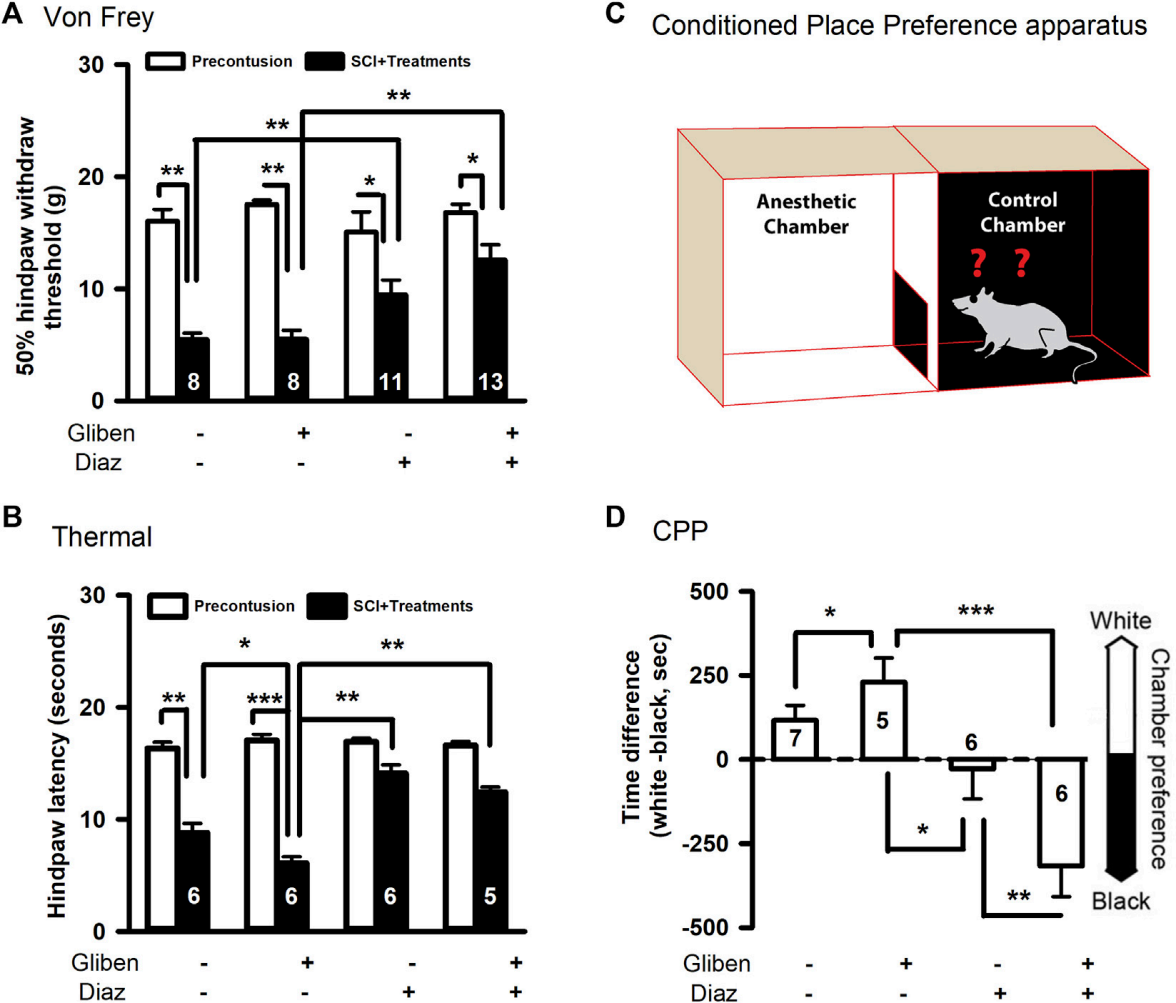
Effects of glibenclamide (Gliben; starting 30 min after contusion for 24 h) and diazoxide (Diaz; starting 3 days post-injury for 10 days) on development of pain behaviors after spinal cord injury (SCI) compared to vehicle control (Veh). **(A)** Mechanical sensitivity of hindpaws was measured by von Frey test 5 weeks after initial traumatic SCI. Animal numbers are indicated on columns. *p < 0.05, **p < 0.01, two-tailed paired *t*-tests (before and after contusion of same group) or two-tailed unpaired t-tests (different treatment groups). **(B)** Thermal sensitivity of hindpaws was measured 5 weeks after SCI. Animal numbers are indicated on columns. *p < 0.05, **p < 0.01, two-tailed unpaired *t*-tests (different treatment groups). **(C)** Conditioned place preference apparatus was used for CPP tests, which include one black and one white chambers. **(D)** Spontaneous pain was evaluated with conditioned place preference (CPP) tests 42 days after contusion. Animal numbers are indicated on columns. *p < 0.05, **p < 0.01, ***p < 0.001, one-way ANOVA followed by Tukey’s *post hoc* test.

At chronic stage of SCI (5 weeks post-contusion), we conducted pain-like mechanical and thermal sensitivity tests. In contrast to a prior study (Redondo-Castro, 2013), glibenclamide (administered 24 h) alone did not exacerbate mechanical hypersensitivity in the von Frey test compared to vehicle control (Figure 3A). However, glibenclamide did significantly worsen thermal (heat) hypersensitivity following SCI compared to vehicle group (Figure 3B). It reported that glibenclamide is still detectable 24 h after a single dose (Coppack et al., 1990), we thus subsequently applied diazoxide starting 3 days post-SCI. Repeated administration of diazoxide or vehicle was then applied for 10 days. The hindpaw withdrawal threshold detected by von Frey test was significantly increased in both diazoxide alone and diazoxide/glibenclamide groups (Figure 3A).

Additionally, diazoxide increased the latency of heat-induced hindpaw withdraw, and glibenclamide did not significantly reverse these effects (Figure 3B). In addition to evoked pain, SCI also induces spontaneous pain in SCI patients, so we performed CPP tests to determine if diazoxide altered the development of SCI-induced spontaneous ongoing pain with or without glibenclamide. SCI animals treated with glibenclamide alone were more inclined to remain in the conditioned analgesic chamber (white chamber) than vehicle control groups, suggesting that early glibenclamide application promotes the development of SCI-induced spontaneous pain. In contrast, diazoxide-treated rats alone or in combination with glibenclamide exhibited no preference for the white chamber, indicating that repeated diazoxide treatment during the acute phase of SCI mitigates later behavioral manifestations of spontaneous pain (Figure 3C). While the combination treatment did not significantly increase the von Frey threshold compared to diazoxide alone (Figure 3A), SCI animals that received the combination treatment were more likely stay in the innately preferred black chamber (Figure 3C). These findings suggest that early, repeated treatment of diazoxide with or without glibenclamide can prevent the development of SCI chronic pain.

### 3.4 combining glibenclamide and diazoxide improves outcomes in motor recovery as compared to glibenclamide alone

**FIGURE 4:**
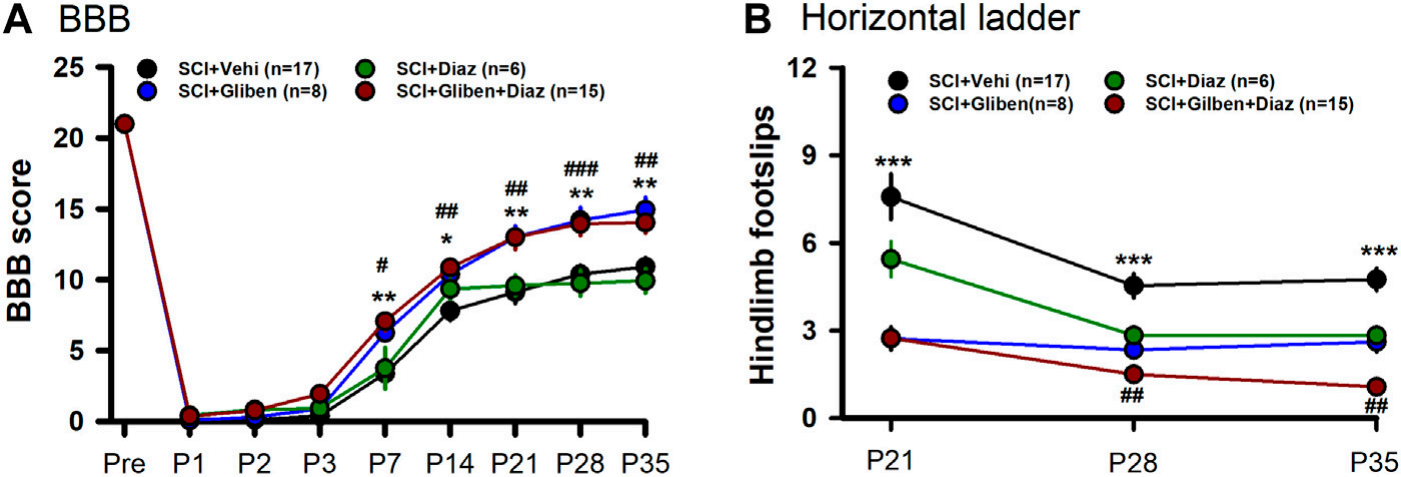
Effects of glibenclamide (Gliben; starting 30 min after contusion for 24 h) and diazoxide (Diaz; starting 3 days post-injury for 10 days) on development of locomotor behaviors after spinal cord injury (SCI) compared to vehicle control (Veh). **(A)** Baso, Beattie, Bresnahan (BBB) locomotor test scoring was performed on days –1, 1, 2, 3, 7, 14, 21, 28, and 35 after contusion. *, #*p* < 0.05, **, ##*p* < 0.01; ###*p* < 0.001; *, ** glibenclamide + diazoxide vs. vehicle + vehicle at same timepoints; #, ##, ### glibenclamide + vehicle vs. vehicle + vehicle at same timepoints; two-tailed unpaired *t*-tests. **(B)** Horizontal ladder test was performed 21, 28, and 35 days after contusion. ***p < 0.001; ##*p* < 0.05; * glibenclamide + vehicle vs. vehicle + vehicle at same timepoints; ## glibenclamide + vehicle vs. glibenclamide + diazoxide at P35; two-tailed unpaired t-tests.

Diazoxide acts as an agonist while glibenclamide acts as an antagonist of SUR subunits. While glibenclamide improves locomotor function following SCI, the impact of diazoxide on glibenclamide-induced beneficial effects is unknown. We thus tested the locomotor function of SCI rats after treatments. Initially, SCI rats received either glibenclamide or vehicle control for 24 h (Simard et al., 2007; Popovich et al., 2012). Subsequently, diazoxide was administered for 10 consecutive days, commencing 3 days post-contusion. The effect of diazoxide on locomotor function post-SCI was assessed using the BBB locomotor test. Consistent with previous reports (Simard et al., 2007; Popovich et al., 2012), glibenclamide significantly improved BBB scores of tested animals (Figure 4A). However, diazoxide alone showed no significant improvement in BBB scores compared to vehicle control at days 7–35 post-SCI, and the combination of glibenclamide/diazoxide did not yield any additional improvement in BBB scores compared to glibenclamide alone.

We also conducted horizontal ladder test to further assess treatment effects. Glibenclamide treatment alone significantly promoted horizontal ladder scores at tested timepoints post-SCI compared to vehicle control (Figure 4B). In contrast to BBB scoring results, SCI rats treated with the combination of glibenclamide/diazoxide exhibited fewer hindlimb footslips compared to rats treated with glibenclamide alone at day 35 post-SCI. These findings suggest that the combination of glibenclamide with diazoxide may enhance locomotor function after SCI.

**FIGURE 5:**
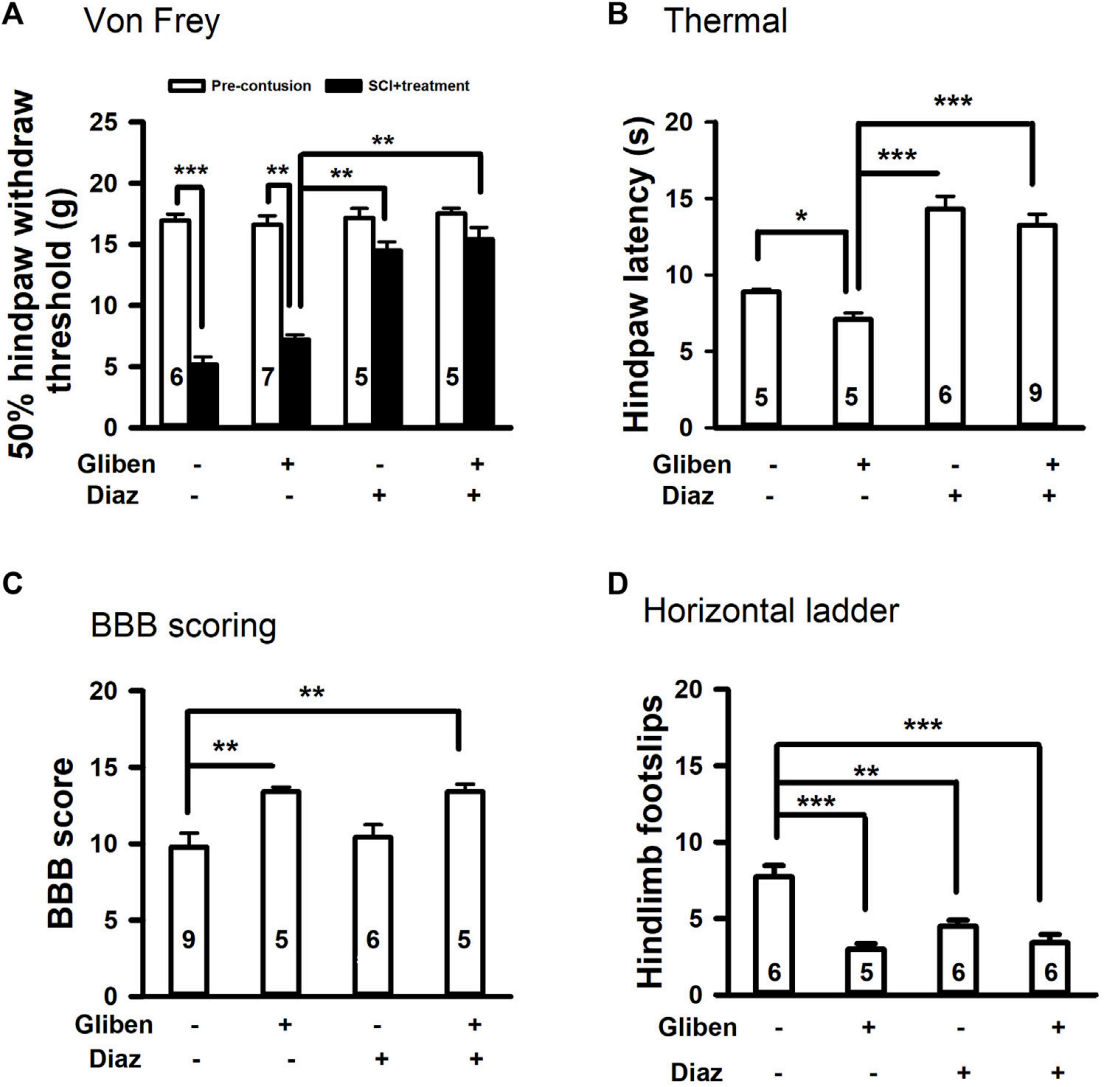
Effects of glibenclamide (Gliben; starting 30 min after contusion for 6 days) and diazoxide (Diaz; starting 6 days post-injury for 7 days) on development of locomotor and sensory behaviors after spinal cord injury (SCI). **(A)** Mechanical sensitivity of hindpaws was measured by von Frey test 35 days after initial traumatic SCI. Animal numbers for each group are indicated on columns. **p < 0.01, ***p < 0.001, two-tailed paired t-tests (before and after treatment of the same group) and two-tailed unpaired *t*-tests (different groups). **(B)** Thermal sensitivity of hindpaws was measured 35 days after contusion. Animal numbers for each group are indicated on columns. *p < 0.05, ***p < 0.001, two-tailed unpaired *t*-tests (different groups). **(C)** Baso, Beattie, Bresnahan (BBB) locomotor testing was performed 42 days after contusion. **p < 0.01, one-way ANOVA followed by Tukey’s *post hoc* test. **(D)** Horizontal ladder test was performed 42 days after contusion. Animal numbers are indicated on columns. **p < 0.01, ***p < 0.001, one-way ANOVA followed by Tukey’s *post hoc* test.

Given that hemorrhage peaks at 3–24 h post-acute SCI and resolves by day 7 (Noble and Wrathall, 1989), it is possible that diazoxide application starting 3 days post-SCI might cause a recurrence of PHN. we thus tested if delayed diazoxide with or without combination of extended glibenclamide would yield better outcomes. Rats received glibenclamide or vehicle for 6 days (day 0—day 5). Subsequently, diazoxide was administered for 7 consecutive days, commencing 6 days post-contusion. Similar to early diazoxide treatment groups, delayed diazoxide treatment significantly prevented the development of mechanical and thermal hypersensitivity even when combined with acute glibenclamide (Figure 5A,B). However, delayed diazoxide treatment following glibenclamide did not further enhance the glibenclamide-induced locomotor improvement detected by BBB scoring and horizontal ladder tests (Figure 5C,D). While delayed diazoxide treatment alone significantly reduced hindpaw foot slips, it did not improve BBB scores after SCI.

### 3.5 Diazoxide alone or in combination with glibenclamide decreases glial cell activation in the spinal cord after SCI

**FIGURE 6:**
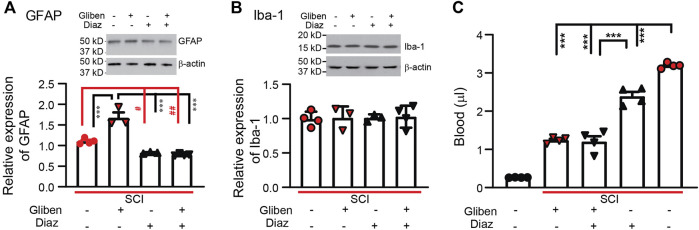
Effects of glibenclamide (Gliben; starting 30 min after contusion for 24 h) and diazoxide (Diaz; starting 3 days post-injury for 10 days) on spinal cord injury (SCI)-induced activation of glial cells and hemorrhage in the spinal cord. **(A)** Quantification of GFAP expression normalized to β-actin in L4/L5 spinal cords. #*p* < 0.05, ##*p* < 0.01, ***p < 0.001, one-way ANOVA followed by Tukey’s *post hoc* test. **(B)** Quantification of IBA-1 expression normalized to β-actin in L4/L5 spinal cords. **(C)** Quantification of extravascular blood in the spinal cord lesion site. ***p < 0.001, one-way ANOVA followed by Tukey’s *post hoc* test. Filled symbols in each column represent individual animals.

Following SCI, both astrocytes and microglia are activated in the spinal cord, contributing to locomotor and sensory dysfunction (Hains and Waxman, 2006; Carlton et al., 2009). To assess the impact of treatments, we quantified GFAP and Iba-1 expression levels in SCI spinal cords. Western blotting showed that glibenclamide alone significantly increased GFAP expression in the spinal cord compared to vehicle-treated controls (Figure 6A). However, diazoxide (starting 3 days after contusion for 10 days) significantly decreased GFAP expression in the spinal cord; combining glibenclamide (starting 30 min after contusion for 24 h) with diazoxide did not significantly further decrease GFAP expression compared to diazoxide alone. Both glibenclamide and diazoxide displayed no obvious effect on Iba-1 expression in the spinal cord following SCI (Figure 6B).

### 3.6 Diazoxide decreases extravascular blood after SCI

While the SUR1 antagonist glibenclamide reduces hemorrhage following SCI (Simard et al., 2007; Simard et al., 2010), we tested whether the SUR1 agonist diazoxide exacerbates hemorrhage following injury. SCI rats received glibenclamide or vehicle for 24 h after contusion, followed by diazoxide for 1 day intraperitoneally starting 3 days post-contusion. Spinal cords encompassing the contusion site were collected on day 4. In contrast to spinal cords from naïve rats, spinal cords subjected to SCI had significantly increased extravascular blood. Similar to previous reports (Simard et al., 2007), acute glibenclamide treatment significantly reduced spinal cord hemorrhage (Figure 6C). Subacute diazoxide treatment alone also significantly reduced spinal cord hemorrhage, although the effect was relatively weaker than that of glibenclamide. Combined glibenclamide/diazoxide treatment did not further reduce spinal cord hemorrhage compared to glibenclamide alone.

### 3.7 Diazoxide does not further preserve spinal cord tissue after glibenclamide treatment

**FIGURE 7:**
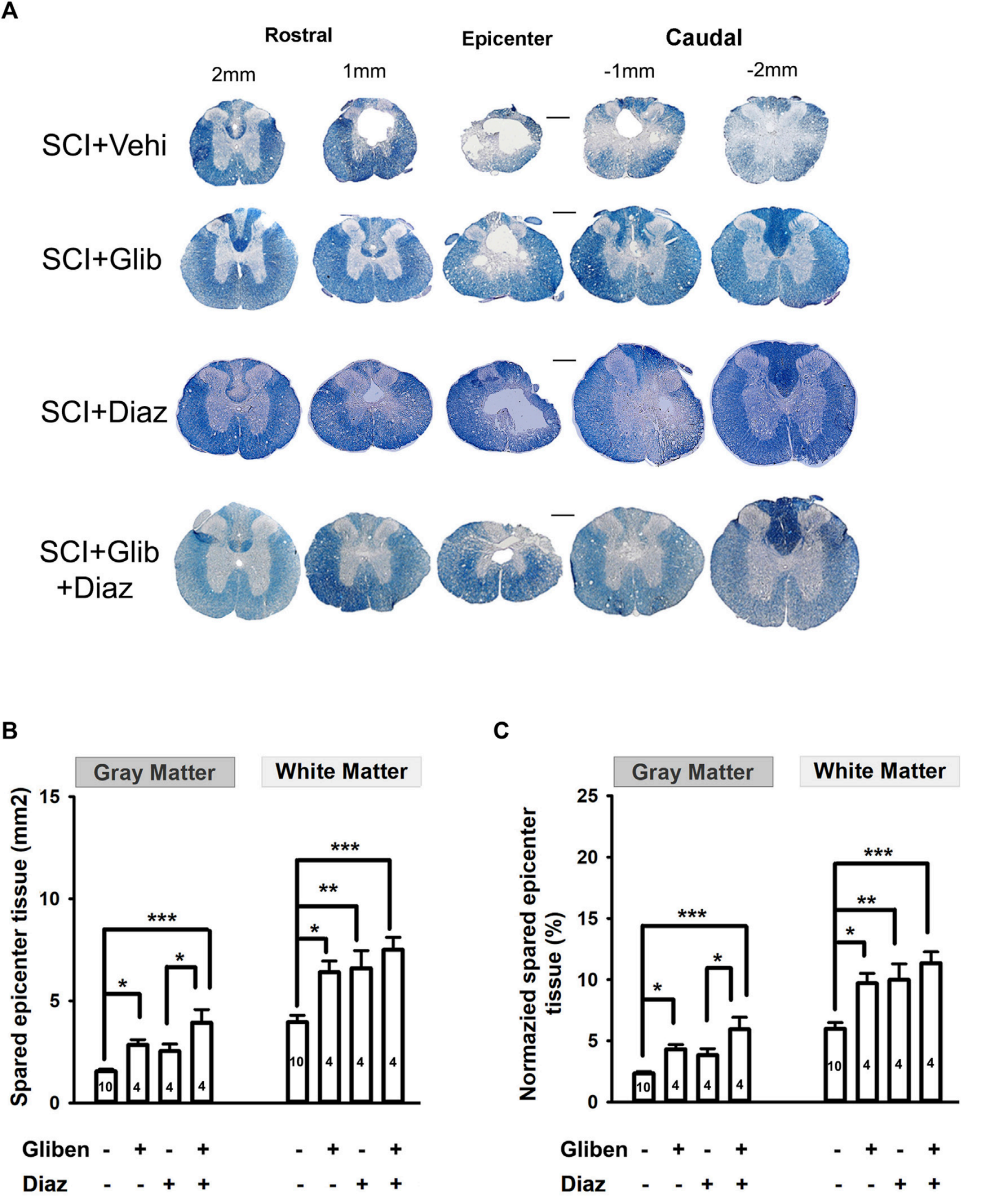
Effects of glibenclamide (Gliben; starting 30 min after contusion for 24 h) and diazoxide (Diaz; starting 3 days post-injury for 10 days) on spinal cord injury (SCI)-induced tissue loss in the spinal cord compared to vehicle control (Veh). **(A)** Representative images showing eriochrome–cyanine staining of spinal cord sections of treated SCI rats. **(B)** Quantification and **(C)** normalization of spared gray matter and white matter of spinal cords at the epicenter of contusion site from glibenclamide and/or diazoxide-treated SCI rats. Animal numbers are indicated on columns. *p < 0.05, one-way ANOVA followed by Tukey’s *post hoc* test. Scale is 400 μm.

Histological evidence was gathered to assess the effect of different treatments by measuring the volume of spared tissue in the spinal cord (gray matter and white matter) by EC staining. Glibenclamide-treated SCI rats had significantly more surviving gray and white matter compared to vehicle-treated rats at P42 (Figure 7B,C). Diazoxide alone only significantly increased the amount of spared white matter, but not gray matter, compared to the vehicle control. However, no further differences in spared gray and white matter were observed from animals treated with the glibenclamide/diazoxide combination, compared to the glibenclamide or diazoxide alone. These findings suggest that glibenclamide, rather than diazoxide, plays a more critical role in reducing degeneration of spinal tissue, especially gray matter, following SCI.

## 4 Discussion

Our findings suggest that timely modulation of SUR subunit activity, through its agonist diazoxide and antagonist glibenclamide, enhances both motor and sensory function following SCI. Diazoxide enhances the activity of K_ATP_ channels expressed in both neurons and glial cells, mitigating both neuronal excitability and glial activation, thereby improving sensory function with or without concurrent glibenclamide administration. When comparing early diazoxide treatment to delayed diazoxide application, similar outcomes were observed for sensory function but not for motor recovery. Early but not delayed diazoxide treatment further enhanced glibenclamide-promoted locomotor function, as evidenced by horizontal ladder test results. Therefore, our results suggest that neuronal hyperexcitation and/or spinal inflammatory activity during the acute stage of SCI are more crucial for development of motor dysfunction than chronic pain induced by SCI.

Administering glibenclamide alone during the acute stage of SCI exacerbates progression of mechanical pain, as assessed by the Randall Selitto test (Redondo-Castro et al., 2013). Although our thermal and CPP tests showed a similar trend, von Frey tests indicated that glibenclamide did not decrease the hindpaw withdrawal threshold. The discrepancy between von Frey and Randall Selitto tests can be attributed to their methodologies—the von Frey test evaluates cutaneous allodynia, likely mediated by Aβ-fibers (Tashima et al., 2018), whereas the Randall Selitto test likely gauges non-cutaneous hypersensitivity transmitted through nociceptors. Thermal hypersensitivity and spontaneous pain are mediated by nociceptors (Yang et al., 2010; Yang et al., 2014). While both cutaneous allodynia and non-cutaneous hypersensitivity arise after SCI (Yang et al., 2010; Yang et al., 2014), glibenclamide might primarily augment the plasticity of nociceptors.

After SCI, both astrocytes and microglial cells become activated not only at the lesion site but also above and below the injury, contributing to development of neurobehavioral dysfunction (Hains and Waxman, 2006; Carlton et al., 2009). K_ATP_ channels are expressed in both astrocytes and microglia (Wu et al., 2011; Rodriguez et al., 2013). While glibenclamide and diazoxide treatment significantly modulated expression of astrocyte marker GFAP in the lesion site, changes in expression of the microglial marker Iba-1 were not significant. Microglia become activated in the spinal cord on day 3 post-SCI (Zhou et al., 2018) and play a crucial role in both initiation and maintenance of SCI-induced chronic pain (Hains and Waxman, 2006; Tan et al., 2009). Iba-1-positive microglia can be classified into two opposing subtypes—classical proinflammatory (M1) and polarized anti-inflammatory alternatives (M2), which have both harmful and beneficial effects in neurodegenerative diseases (Jiang et al., 2020; Guo et al., 2022). While we observed a protective effect of diazoxide in development of chronic pain, it is possible that diazoxide enhances transition of microglia from M1 to M2 without affecting Iba-1 expression (Rodriguez et al., 2013). This possibility requires further evaluation.

Both glibenclamide and diazoxide decreased extravascular blood in the spinal cord. Hemorrhage peaks at 3–24 h after acute SCI and resolves by day 7 (Noble and Wrathall, 1989). Acute administration of glibenclamide reduces secondary hemorrhage in the spinal cord after SCI by preventing fragmentation of spinal cord capillaries through TRPM4 ion channels (Gerzanich et al., 2009). Absorption of hematomas after hemorrhage primarily relies on phagocytes’ ability to engulf red blood cells (Fu et al., 2023). Activation of both K_ATP_ channels and TRPM4 channels contributes to enhanced activity of monocytes/macrophages (Serafini et al., 2012; Ling et al., 2013), so it is possible that diazoxide mitigates extravascular blood by promoting hematoma absorption. However, the impact of delayed administration of diazoxide on this effect may not be significant in combination treatment, as the pool of extravascular blood is already limited by acute glibenclamide application.

Heightened neuronal excitability and activation of spinal cord glial cells are pivotal in onset of dysfunction following SCI (Lee et al., 2003; Hains and Waxman, 2006; Tan et al., 2009). In this regard, targeting neuronal hyperexcitability with retigabine, a selective KCNQ ion channel activator, improves both motor and sensory dysfunction after SCI (Wu et al., 2020). Intraperitoneal injection of minocycline, a nonspecific microglial inhibitor (Tikka et al., 2001), reduces lesion size and hyperexcitability of spinal neurons in a rodent SCI model. Diazoxide enhances the activity of K_ATP_ channels expressed in both neurons and glial cells, mitigating both neuronal excitability and glial activation, thereby improving sensory function with or without concurrent acute glibenclamide. When comparing the early application of diazoxide to its delayed application with or without glibenclamide, similar outcomes are observed for sensory function but not for motor function. Early diazoxide administration, but not delayed administration, further enhances glibenclamide-promoted locomotor function, as evidenced by horizontal ladder tests. This study suggests that neuronal hyperexcitation and/or spinal inflammatory activity during the early subacute stage are more crucial for the development of motor dysfunction than the pain chronification induced by SCI.

Our previous study demonstrated that decreasing neuronal hyperexcitability with retigabine starting 3 h post-SCI results better outcomes, particularly in locomotor function, compared to a delayed administration (Wu et al., 2020). This emphasizes the critical role of the timing of pharmacological intervention following SCI, prompting further investigation into the optimization of therapeutic windows for glibenclamide and diazoxide following SCI. Glibenclamide and diazoxide interact with the SUR subunit but in opposite ways. Given this dichotomy, our study avoided their simultaneous application to prevent possible antagonistic interactions. K_*ATP*_ and TRPM4 ion channels exhibit varying sensitivities to these agents across different tissues (Stephan et al., 2006; Kovács et al., 2022). This raises the question of whether the sensitivity ratio of diazoxide to glibenclamide is higher in neurons and glia compared to capillary endothelial cells. If this is the case, diazoxide could be administered at lower doses initially in conjunction with glibenclamide, and then increased when administered alone. Alternatively, an activator of different K^*+*^ channels, such as retigabine (Wu et al., 2020), could be used in the presence of glibenclamide. This approach may enhance overall neuroprotection and provide a more effective therapeutic strategy post-SCI.

## 5 Conclusion

While inhibiting SUR subunits at the acute stage improves locomotor function, it exacerbates the development of chronic pain following SCI (Redondo-Castro et al., 2013). We found that activating SUR subunits at the subacute stage can mitigate the development of SCI-induced chronic pain. Combining glibenclamide at the acute stage and diazoxide at the subacute stage can improve both locomotor and sensory function following SCI. The possible clinical relevance of these studies is enhanced by our choice of SUR modulators approved by the FDA for other uses. In the future, we need to optimize the time window for diazoxide application so that the beneficial effects of the combined treatment strategy can be maximized.

## 6 Transparency, rigor, and reproducibility summary

An individual not directly engaged in the experiment’s execution prepared reagents and labeled them with numerical identifiers. Animals were randomly allocated to different groups. All behavior evaluations and histological examinations were carried out in a blinded manner. Un-blinding was done after conclusion of data collection, data input, and database formatting. At least 5 animals were used for behavioral tests in each group and at least 3 replicates in each group were used for Western blot and extravascular blood analysis. Experiments were performed with male and female animals. A total of 101 rats were used in the study, 6 were dead due to surgery, 3 were excluded from analysis because their BBB scores are more than one at day one.

## Data Availability

The original contributions presented in the study are included in the article/Supplementary Material, further inquiries can be directed to the corresponding authors.

## Abbreviations

BBB, Baso, Beattie, Bresnahan; CPP, conditioned place preference; EC, eriochrome cyanine; K_ATP_, ATP-gated potassium channels; PHN, progressive hemorrhagic necrosis; SCI, spinal cord injury; SUR, regulatory sulfonylurea; TRPM4, transient receptor potential cation channel subfamily melastatin-4.
